# Massive Lymphedema in the Calf Complicated by Severe Skin Fibrosclerosis

**DOI:** 10.70352/scrj.cr.25-0375

**Published:** 2025-08-26

**Authors:** Kotaro Suehiro, Hiroyuki Takasu, Seiko Fujino, Takasuke Harada, Makoto Samura, Yuriko Takeuchi, Takahiro Mizoguchi, Hiroshi Kurazumi, Ryo Suzuki, Kimikazu Hamano

**Affiliations:** 1 Department of Surgery and Clinical Science, Yamaguchi University Graduate School of Medicine, Ube, Yamaguchi, Japan; 2 Department of Plastic Surgery, Yamaguchi University Hospital, Ube, Yamaguchi, Japan; 3 Department of Nursing, Yamaguchi University Hospital, Ube, Yamaguchi, Japan

**Keywords:** lymphedema, elephantiasis, reductive surgery

## Abstract

**INTRODUCTION:**

Lymphedema is generally managed with conservative therapy. However, in cases of severe fibrosclerotic lymphedema, debulking surgery is required, although rarely. We present a case of massive lymphedema in the left calf complicated by severe skin fibrosclerosis that was successfully managed with debulking surgery.

**CASE PRESENTATION:**

A 58-year-old woman presented to our clinic with bilateral leg swelling, which was particularly massive in the left calf. She could hardly walk independently and experienced cellulitis 2 to 4 times a year. The patient was admitted, and aggressive decongestion with compression therapy was attempted initially. However, this was unsuccessful due to severe skin hardening caused by abnormal dermal thickening. We then performed partial subcutaneous tissue resection and wrapping with the redundant skin, but this resulted in extensive skin necrosis. Finally, resection of the whole skin and subcutaneous tissue down to the deep fascia in the left calf was performed, followed by split-thickness skin grafting harvested from the left thigh. At present, one year after the surgery, the patient is capable of performing light exercise and has not experienced a recurrence of cellulitis.

**CONCLUSIONS:**

When preoperative conservative therapy is unsuccessful due to severe skin fibrosclerosis, earlier surgical intervention, including debulking, is beneficial in the management of massive lymphedema.

## INTRODUCTION

Lymphedema is generally managed using complex decongestive therapy, including compression therapy, manual lymphatic drainage, meticulous skin care, and muscle pumping exercises. However, in cases of severe fibrosclerotic lymphedema, microsurgical procedures such as lymphovenous anastomosis are not indicated, and debulking surgery, namely resection of excess skin and subcutaneous tissue, may be required, although the indication is rare because the procedure is often associated with complications such as worsening of lymphedema, scarring, infection, and poor wound healing.^1)^ In this report, we present a case of massive lymphedema limited to the left calf, which was resistant to conservative therapy due to severe skin hardening caused by extensive skin fibrosis, and benefitted from debulking surgery.

## CASE PRESENTATION

A 58-year-old woman presented to our clinic with bilateral leg swelling that was particularly severe in the left calf. At the age of 32 years, she had undergone a hysterectomy and pelvic node dissection, followed by radiation therapy for cervical cancer. At the age of 38 years, her legs began to swell, suggesting the onset of lymphedema. She visited several clinics but received no proper treatment. Her legs, especially the left calf, swelled progressively and were deformed. She experienced her first episode of cellulitis in the left leg at 48 years of age, followed by repetitive cellulitis 2 to 4 times per year.

**Fig. 1:**
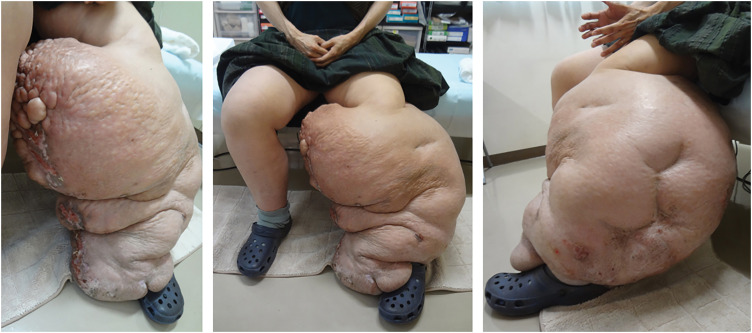
The left leg at the initial visit. The left calf is disproportionately enlarged and severely disfigured. Multiple active/healed ulcers and warty nodules are observed on the medial side.

During her initial visit to our clinic, bilateral leg swelling was noted; however, the left calf was disproportionately enlarged, with a maximum circumference of 128 cm, where multiple active/healed ulcers and warty nodules were observed (**Fig. 1**), which was classified as stage III according to the International Society of Lymphology classification.^1)^ Her height and weight were 167 cm and 112.5 kg (body mass index 40 kg/m^2^), respectively, although her upper body seemed thin, suggesting that most of the excess weight was derived from the legs. Her serum albumin level was 2.6 g/dL; chest radiography and echocardiography suggested mild heart failure, possibly due to increased circulating blood volume. She demonstrated severe gait disturbance due to leg heaviness, all of which further exacerbated the leg edema. Malignancies including recurrent cervical cancer were ruled out. Duplex venous scan revealed no abnormalities, and symptoms suggestive of chronic venous insufficiency were not confirmed. In lymphangioscintigraphy, no lymphatics were visualized in both legs.

**Fig. 2:**
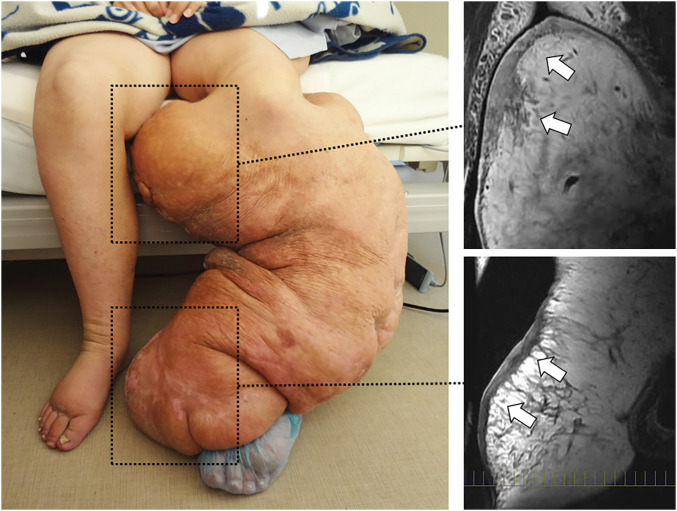
The left leg following aggressive decongestion. In the upper and lower medial calf, where hard and round protrusions were present (dotted rectangles), shrinkage could not be achieved with compression therapy. MRI revealed severe skin thickening (white arrows) in these areas.

She was admitted, and aggressive decongestion through compression therapy was attempted using commercially available back braces of various sizes (22 cm wide, 118–148 cm long) and furosemide (20 mg daily). During the first 4 weeks, her weight was reduced by 22 kg, of which 17 kg was estimated to have been from the left calf. However, the volume reduction plateaued thereafter because the large protruding nodules in the upper and lower medial calf were unresponsive to the compression therapy (**Fig. 2**). Since these nodules were hard and round in shape, the locally applied bandages easily slipped off, as if applied to a helmet. MRI revealed a severely thickened dermis with a thickness of 5–17 mm in these areas. Accordingly, we decided to terminate conservative therapy, and debulking surgery was planned with the aid of plastic surgeons.

**Fig. 3:**
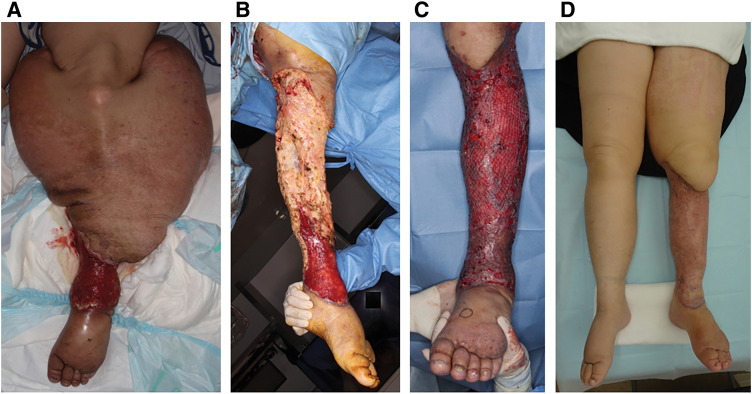
Surgeries for the left calf. (**A**) Partial subcutaneous tissue resection (lower calf) and wrapping with redundant skin resulted in skin necrosis. (**B**) Resection of the entire skin and subcutaneous tissue down to the deep fascia was performed. (**C**) Split-thickness skin grafting was performed after achieving good stabilization of the wound bed. (**D**) The legs one year after debulking surgery.

Initially, partial subcutaneous tissue resection and wrapping with redundant skin were performed; however, this resulted in extensive skin necrosis followed by wound infection (**Fig. 3A**). Accordingly, resection of the entire skin and subcutaneous tissue down to the deep fascia of the entire left calf was performed (**Fig. 3B**). This time, the affected limb had been prepared with elevation and multilayer bandaging overnight to minimize the intra- and postoperative fluid loss. Histological samples revealed thickened skin with extensive fibrosis and edema, some ectatic lymphatics, and scattered multinucleated cells, which are typical of severe lymphedema, but no evidence of malignancy. After achieving good granulation of the wound bed, which required approximately a month, split-thickness skin grafting, i.e., modified Charles procedure,^2)^ was performed; the graft was harvested from the left thigh using a dermatome (**Fig. 3C**). Approximately a year after the surgery, no weeping or skin breaks were noticed (**Fig. 3D**). At the latest follow-up, the patient weighed 67 kg (body mass index 24 kg/m^2^). She can now perform light exercises such as jogging and ping pong. In addition, she has not experienced cellulitis since the surgery. Although no edema is confirmed in the surgical site, the patient is currently using an ordinary compression garment to manage edema in the thigh and foot. However, persistent foot swelling is poorly managed at present, which is a common problem following the Charles procedure.

## DISCUSSION

### Indication of debulking surgery for advanced lymphedema

Debulking surgeries such as the Charles procedure, which includes a radical circumferential excision of the lymphedema tissue down to the deep fascia and reconstruction with skin grafts, have been performed for more than a hundred years. Previously, the procedure was often complicated by infection and/or poor wound healing, occasionally resulting in major amputation.^3)^ However, the outcome has improved due to modifications in procedures and perioperative care; currently, debulking surgery has become an important option to manage severe fibrosclerotic lymphedema.^2,4)^ In the initial stage of treatment for massive lymphedema, edema reduction, mainly using compression therapy, is indispensable to prevent poor postoperative wound healing and lymphorrhea.^5)^ However, effective decongestion may be hindered when severe skin hardening occurs, as in this case. In such cases, surgical intervention at an earlier stage of treatment is essential. However, the surgical team should prepare for a larger fluid and blood loss. In the present case, preoperative leg elevation and aggressive overnight bandaging seemed particularly effective.

First, partial resection of the subcutaneous tissue and wrapping with redundant skin were attempted; however, this resulted in extensive skin necrosis. This was possibly due to the poor blood supply to the skin with elephantiasis. Other studies have stated that this procedure can result in skin necrosis.^6)^ Therefore, we believe that thorough tissue resection and grafting with healthier skin should be planned when initiating treatment.

### Causes of massive swelling limited to the calf

In the current case, swelling of the left lower calf was disproportionate despite the presence of lymphedema in both legs. Previous reports have stated that massive localized lymphedema is frequently associated with obesity^7,8)^; however, this was not the case in the current patient. Lu et al. reported that other possible factors in the development of localized elephantiasis include tissue injury that blocks the lymphatic pathway, underlying chronic inflammatory diseases that cause increased expression of vascular endothelial growth factor, and bacterial infection/cellulitis.^9)^ The patient had experienced frequent episodes of cellulitis, which might have caused a localized obstruction of lymphatic drainage. Additionally, in later stages, excessive pendulous skin and subcutaneous tissue may further exacerbate local lymph congestion. However, the mechanism underlying the excessive local swelling remains unclear and should be clarified in future studies.

## CONCLUSIONS

Here, we report a case of massive lymphedema in the left calf that was successfully managed using a modified Charles procedure. When preoperative conservative therapy is unsuccessful owing to severe skin fibrosclerosis, early surgical intervention using a debulking procedure would be beneficial to manage such cases.

## DECLARATIONS

### Availability of data and materials

The datasets supporting the conclusions of this article are included within the article.
